# Supplementary material for: Italian cross-cultural adaptation of the Quality of Communication questionnaire and the 4-item advance care planning engagement questionnaire
Source: PLoS One. 2023 Mar 23;18(3):e0282960. doi: 10.1371/journal.pone.0282960 (PMC10035811; doi:10.1371/journal.pone.0282960)
Supplement: S1 File — (PDF) [file pone.0282960.s001.pdf]

Translation report QOC from US English to Italian

S1 File. Italian QOC Translation grid

COMMENTS/QUESTIONS MADE BY:		
PP (Consultant Italian translator 1)	AS (Researcher 1)	MAF (Lay person)
FB (Consultant Italian translator 2)	SV (Researcher 2)	PK (Expert patient)
LR (Consultant backward translator)	AG (Researcher 3)	*RE/EN (scale authors)*

TITLE

ORIGINAL ENGLISH WORDING	COMMENTS/QUESTIONS
Quality of Communication Questionnaire	
**ITALIAN TRANSLATION 1**	
Questionario sulla qualità della comunicazione	
**ITALIAN TRANSLATION 2**	
Questionario sulla qualità della comunicazione	
**RECONCILED ITALIAN TRANSLATION**	
Questionario sulla qualità della comunicazione	
**BACKWARD TRANSLATION**	
Quality of Communication Questionnaire	
**FINAL ITALIAN VERSION**	
Questionario sulla qualità della comunicazione	

SUB-TITLE

ORIGINAL ENGLISH WORDING	COMMENTS/QUESTIONS
Doctor Communication	
**ITALIAN TRANSLATION 1**	
Comunicazione con il medico	
**ITALIAN TRANSLATION 2**	
Comunicazione del medico	
**RECONCILED ITALIAN TRANSLATION**	

Translation report QOC from US English to Italian

Comunicazione con il medico	
**BACKWARD TRANSLATION**	
Communicating with the doctor	
**PRE-FINAL ITALIAN VERSION**	The competences of the doctor is the focus here!<br>Doctor communication skils (whether it is the doctor that is assessed In the Italian version could be: Competenze comunicative, capacità di comunicare, Capacità nel comunicare<br>La qualità comunicativa del medico?
Capacità di comunicazione del medico	
**FINAL ITALIAN VERSION**	
Capacità di comunicazione del medico	

INTRODUCTION *(first part)*

ORIGINAL ENGLISH WORDING	COMMENTS/QUESTIONS
We would like to know, in as much detail as possible, how good the doctor taking care of your [~~respiratory/lung~~] problems is at talking with you about your illness and the types of care that you would want if you became sicker or too sick to speak for yourself.<br>We know that many people think very highly of their doctors. To help us improve communication between doctors and their patients, please be critical.	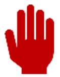 To be clarified what should replace the original "respiratory/lung" **QUERY 1**<br>*ANSWER 1 -- The backward translation* that you have in the table is acceptable. It would be acceptable to identify [a particular condition] or [activity, e.g. Advance Care Planning] or reference the [patient's care].*<br>The sentence is very long
**ITALIAN TRANSLATION 1**	With this questionnaire, the back-translation will be a challenge<br>... a compromise between literal translation and conceptual equivalence
Riguardo al medico che la cura, vorremmo sapere, nel modo più dettagliato possibile, quanto sia bravo a parlare con lei della sua malattia e del tipo di cure che vorrebbe ricevere se dovesse aggravarsi o fosse troppo malato per esprimersi autonomamente.<br>Sappiamo che molte persone hanno grandissima stima per il loro medico. La preghiamo di assumere un atteggiamento critico, per aiutarci a migliorare la comunicazione tra i medici e i pazienti.	
**ITALIAN TRANSLATION 2**	

Vorremmo sapere, con più dettagli possibili, quanto sia bravo il medico che si prende cura dei suoi problemi a parlare con lei della sua malattia e dei tipi di assistenza che lei vorrebbe nel caso lei peggiorasse o fosse troppo malato da parlare per sé stesso.<br>Sappiamo che molte persone hanno una grande considerazione del proprio medico. Per aiutarci a migliorare la comunicazione fra i medici e i loro pazienti, per favore sia obiettivo.	
**RECONCILED ITALIAN TRANSLATION**	Assistenza o cura?
Vorremmo sapere, nel modo più dettagliato possibile, come lei valuta la capacità del medico che l'ha in cura nel parlare con lei della sua malattia e del tipo di assistenza che vorrebbe ricevere se dovesse aggravarsi o fosse troppo malato per esprimersi autonomamente.<br>Sappiamo che molte persone hanno una grande considerazione per il proprio medico. La preghiamo di essere obiettivo per aiutarci a migliorare la comunicazione tra i medici e i loro pazienti.	
**BACKWARD TRANSLATION**	
We would like to know in as much detail as possible how well you think the doctor treating you for (...) talks with you about your illness and the type of medical care you would like to receive if your condition worsened or you were too sick to speak for yourself.<br>We know that many people think very highly of their doctor. Please try to be as objective as possible to help us improve communications between doctors and their patients.	
**PRE FINAL ITALIAN VERSION**	Better to reverse the order of the last sentence (agreed)<br>'Migliorare la qualità...' (agreed)<br>Text to be added in order to disambiguate who is the physician whose competences are assessed (adjunct originated from the Translation Panel)
Vorremmo sapere, nel modo più dettagliato possibile, come lei valuta la capacità del medico che l'ha in cura nel parlare con lei della sua malattia e del tipo di assistenza che vorrebbe ricevere se dovesse aggravarsi o fosse troppo malato per esprimersi autonomamente.<br>Sappiamo che molte persone hanno una grande considerazione per il proprio medico. Per aiutarci a migliorare la qualità della comunicazione medico-paziente, la preghiamo di essere obiettivo.	
**FINAL ITALIAN VERSION**	*It would be acceptable to identify [a particular condition] or [activity, e.g. Advance Care Planning] or reference the [patient's care].*
Vorremmo sapere, nel modo più dettagliato possibile, come lei valuta la capacità del medico che l'ha in cura **[per/presso] [inserire patologia / setting di cura]** nel parlare con lei della sua malattia e del tipo di assistenza che vorrebbe ricevere se dovesse aggravarsi o fosse troppo malato per esprimersi autonomamente.	

Sappiamo che molte persone hanno una grande considerazione per il proprio medico. Per aiutarci a migliorare la qualità della comunicazione medico-paziente, la preghiamo di essere obiettivo.	

INTRODUCTION *(second part)*

ORIGINAL ENGLISH WORDING	COMMENTS/QUESTIONS
Using the following scale, where “0” is the worst you could imagine, and “10” is the best you could imagine, please select the best number for each statement.	electronic version vs printed one (“select” for interview, and e-versions, “tick/circle” for printed) **QUERY 2** *ANSWER -- It makes sense to me to have the instruction correspond with the administration method; no concerns that they are different between two versions in use at the same time*
**ITALIAN TRANSLATION 1**	
Sulla scala che segue, dove "0" rappresenta il peggio che riesca a immaginare, e "10" il meglio che possa immaginare, scelga il numero che descrive il livello di ciascuna voce.	
**ITALIAN TRANSLATION 2**	
Usando la seguente scala dove “0” è il peggiore che lei possa immaginare e “10” è il migliore che lei possa immaginare, per favore scelga il numero più adatto per ogni affermazione.	
**RECONCILED ITALIAN TRANSLATION**	
Sulla scala che segue, dove "0" rappresenta il peggio che possa immaginare e “10” il meglio che possa immaginare, per ciascuna affermazione segni il numero che corrisponde alla sua valutazione.	
**BACKWARD TRANSLATION**	
Please answer each of the following questions using a scale form “0” = worst imaginable to “10” = best imaginable.	
**PRE FINAL ITALIAN VERSION**	
Sulla scala che segue, dove "0" rappresenta il peggio che possa immaginare e “10” il meglio che possa immaginare, per ciascuna affermazione scelga il numero che corrisponde alla sua valutazione.	*Suggestion to add this statement at the end of the instructions: You may also check the box, 'I do not know.'" OR "If you don't know how to rate your doctor on an item, please check the box, 'I don't know'. If you can't rate an item because your doctor has*

Risponda ‘non valutabile’, se quell’azione o comportamento non ha avuto luogo. Risponda ‘non saprei’, se è incerto ~~nell’esprimere un~~ sul giudizio.	*not talked with you about it, please check the box, 'The doctor did not do this'.*
**FINAL ITALIAN VERSION**	
Sulla scala che segue, dove "0" rappresenta il peggio che possa immaginare e “10” il meglio che possa immaginare, per ciascuna affermazione **[scelga]/[selezioni]** il numero che corrisponde alla sua valutazione.<br>Risponda ‘non valutabile’, se quell’azione o comportamento non ha avuto luogo. Risponda ‘non saprei’, se è incerto sul giudizio.	Depending on the format of the questionnaire (paper or electronic) the italian verb should be different

GENERAL STATEMENT BEFORE ITEMS 1- 17

**ORIGINAL ENGLISH WORDING**	**COMMENTS/QUESTIONS**
When talking with Doctor (X) about important issues like becoming very ill, how good is he/she at:	
**ITALIAN TRANSLATION 1**	If the name of a doctor is specified, the sentence should be revised accordingly (bravo/brava)
Quando parla con il Dr. (X) di problemi importanti, come l'eventualità di aggravarsi seriamente, quanto lo giudica bravo nei seguenti aspetti?	
**ITALIAN TRANSLATION 2**	
Quando parla con il Dottor (X) di questioni importanti come il diventare molto malato, quanto lui/lei è in grado di :	
**RECONCILED ITALIAN TRANSLATION**	Dr. X /your doctor/this doctor. Need to increase consistency along the questionnaire. To do this, in the reconciled Italian translation 2 we used ‘il suo medico’ (your doctor) in all istances
Quando parla con il Dottor (X) di problemi importanti, come l'eventualità di un grave peggioramento, quanto lo valuta capace di:	
**BACKWARD TRANSLATION**	
When you talk with Doctor (X...) about important things like the chances of your condition worsening, how good do you think he/she is at:	
**PRE FINAL ITALIAN VERSION**	‘Issues’ is...: problemi? Aspetti? Cose? Questioni!
Quando parla con il Dottor (X) di questioni importanti, come l'eventualità di un grave peggioramento della sua malattia, quanto lo valuta capace di:	

Translation report QOC from US English to Italian

FINAL ITALIAN VERSION	
Quando parla con il suo medico di questioni importanti, come l'eventualità di un grave peggioramento della sua malattia, quanto lo valuta capace di:	

Anchor # 1

ORIGINAL ENGLISH WORDING	COMMENTS/QUESTIONS
The very worst I could imagine	
**ITALIAN TRANSLATION 1**	
Il peggio che posso immaginare	
**ITALIAN TRANSLATION 2**	
Il peggiore che io possa immaginare	
**RECONCILED ITALIAN TRANSLATION**	
Il peggio che possa immaginare	
**BACKWARD TRANSLATION**	
Worst imaginable	
**FINAL ITALIAN VERSION**	
Il peggio che possa immaginare	

Anchor # 2

ORIGINAL ENGLISH WORDING	COMMENTS/QUESTIONS
The very best I could imagine	
**ITALIAN TRANSLATION 1**	
Il meglio che posso immaginare	
**ITALIAN TRANSLATION 2**	
Il migliore che io possa immaginare	
**RECONCILED ITALIAN TRANSLATION**	
Il meglio che possa immaginare	
**BACKWARD TRANSLATION**	
Best imaginable	

**FINAL ITALIAN VERSION**	
Il meglio che possa immaginare	

POSSIBLE REPLY # 3

**ORIGINAL ENGLISH WORDING**	**COMMENTS/QUESTIONS**
Didn’t Do	
**ITALIAN TRANSLATION 1**	
Mai fatto	
**ITALIAN TRANSLATION 2**	
Non lo ha fatto	
**RECONCILED ITALIAN TRANSLATION**	
Non lo ha fatto	To disambiguate with the scale Author (**QUERY 3**)<br>*ANSWER -- This is meant to indicate "My clinician has not done this." / "The doctor did not do this."*
**BACKWARD TRANSLATION**	
He/she never has	
**PRE FINAL ITALIAN VERSION**	The Panel considered the answer of the Author to Query 3 above and, in cooherence with the added instructions (following the Author’s suggestion, see page 4) agreed to replace ‘non lo ha fatto’(didn’t do) with ’non valutabile’ (not applicable). Considering e.g. item # 2 (‘Looking you in the eye’), the answer ‘didn’t do’ could also correspond to a score of ‘0’ on the numeric scale (‘The very worst I could imagine’). The answer ‘not applicable’ instead is a viable option in case of a blind patient or telephone consultation.
Non valutabile	
**FINAL ITALIAN VERSION**	
Non valutabile	

POSSIBLE REPLY # 4

ORIGINAL ENGLISH WORDING	COMMENTS/QUESTIONS
Don't Know	
**ITALIAN TRANSLATION 1**	
Non so	
**ITALIAN TRANSLATION 2**	
Non so	
**RECONCILED ITALIAN TRANSLATION**	Same as above **(QUERY 3)** *ANSWER -- This is meant to indicate "I do not know."*
Non saprei	
**BACKWARD TRANSLATION**	
Not sure	
**FINAL ITALIAN VERSION**	We also added instructions, as suggested by the Scale Author
Non saprei	

ITEM # 1

ORIGINAL ENGLISH WORDING	COMMENTS/QUESTIONS
Using words that you can understand	
**ITALIAN TRANSLATION 1**	
Usare parole che lei possa capire	
**ITALIAN TRANSLATION 2**	
Usare parole che lei può comprendere	
**RECONCILED ITALIAN TRANSLATION**	
Usare parole che lei può comprendere	
**BACKWARD TRANSLATION**	
Using words you understand	
**FINAL ITALIAN VERSION**	
Usare parole che lei può comprendere	

Translation report QOC from US English to Italian

ITEM # 2

ORIGINAL ENGLISH WORDING	COMMENTS/QUESTIONS
Looking you in the eye	
**ITALIAN TRANSLATION 1**	
Guardarla negli occhi	
**ITALIAN TRANSLATION 2**	
Guardarla negli occhi	
**RECONCILED ITALIAN TRANSLATION**	
Guardarla negli occhi	
**BACKWARD TRANSLATION**	
Looking you in the eye	
**FINAL ITALIAN TRANSLATION**	
Guardarla negli occhi	

ITEM # 3

ORIGINAL ENGLISH WORDING	COMMENTS/QUESTIONS
Including your loved ones in decisions about your illness and treatment	
**ITALIAN TRANSLATION 1**	
Coinvolgere i suoi cari nelle decisioni che riguardano la sua malattia e la cura	
**ITALIAN TRANSLATION 2**	
Includere i suoi cari nelle decisioni riguardo alla sua malattia e terapia	
**RECONCILED ITALIAN TRANSLATION**	
Coinvolgere i suoi cari nelle decisioni che riguardano la sua malattia e le cure	
**BACKWARD TRANSLATION**	
Involving your loved ones in decisions concerning your illness and treatment	
**PRE FINAL ITALIAN VERSION**	*We believe the right choice would be 'terapie" (treatment, more specific) in items # 3/4/13.*
Coinvolgere i suoi cari nelle decisioni che riguardano la sua malattia e le terapie	
**FINAL ITALIAN VERSION**	
Coinvolgere i suoi cari nelle decisioni che riguardano la sua malattia e le terapie	

Translation report QOC from US English to Italian

ITEM # 4

ORIGINAL ENGLISH WORDING	COMMENTS/QUESTIONS
Answering all your questions about your illness and treatment	
**ITALIAN TRANSLATION 1**	
Rispondere a tutte le sue domande riguardo alla sua malattia e alle cure	
**ITALIAN TRANSLATION 2**	
Rispondere a tutte le sue domande in merito alla sua malattia e terapia	
**RECONCILED ITALIAN TRANSLATION**	
Rispondere a tutte le sue domande riguardo alla sua malattia e alle cure	
**BACKWARD TRANSLATION**	
Answering all your questions about your illness and medical care	
**PRE FINAL ITALIAN VERSION**	„Cure“ is more comprehensive than „treatment“, should we go back to „ terapie“?<br>Here ‘treatment’ appears for the first time. Before the term was “care”<br>This was the case also for Item # 3 and item #13<br>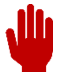 To be clarified with the Author: ‘’treatment’’ appears for item # 3, 4 and 13. In the introduction, the term was “medical care”. The Italian translation "assistenza“ ("care" in English) is more comprehensive than "terapie". Should we use "assistenza" in the introduction and 'terapie" in items # 3/4/13 or we can use " assistenza" consistently along the questionnaire?**QUERY 4**<br>*ANSWER -- We believe the right choice would be to use "assistenza" (care-- more comprehensive) in the introduction and 'terapie" (treatment, more specific) in items # 3/4/13.*
Rispondere a tutte le sue domande riguardo alla sua malattia e alle cure	
**FINAL ITALIAN VERSION**	
Rispondere a tutte le sue domande riguardo alla sua malattia e alle terapie	

ITEM # 5

ORIGINAL ENGLISH WORDING	COMMENTS/QUESTIONS
Listening to what you have to say	
ITALIAN TRANSLATION 1	
Ascoltare le cose che ha da dire	
ITALIAN TRANSLATION 2	
Ascoltare quello che lei ha da dire	
RECONCILED ITALIAN TRANSLATION	
Ascoltare le cose che lei ha da dire	
BACKWARD TRANSLATION	
Listening to what you have to say	
FINAL ITALIAN VERSION	
Ascoltare le cose che lei ha da dire	

ITEM # 6

ORIGINAL ENGLISH WORDING	COMMENTS/QUESTIONS
Caring about you as a person	
ITALIAN TRANSLATION 1	
Prendersi cura di lei come persona	
ITALIAN TRANSLATION 2	
Prendersi cura di lei come persona	
RECONCILED ITALIAN TRANSLATION<br>Prendersi cura di lei come persona	An important issue (all over the questionnaire) is whether to use the second person instead, to be more direct and confidential.<br>Use of the third person seems more respectful and delicate in view of these sensible contents<br>I also arrived at the third person<br>The Panel agrees on the use of the third person (this issue does not apply to the English language)

BACKWARD TRANSLATION	
Caring for you as a person	
**FINAL ITALIAN VERSION**	
Prendersi cura di lei come persona	

ITEM # 7

ORIGINAL ENGLISH WORDING	COMMENTS/QUESTIONS
Giving you his/her full attention	
**ITALIAN TRANSLATION 1**	
Prestarle piena attenzione	
**ITALIAN TRANSLATION 2**	
Prestarle la sua piena attenzione	
**RECONCILED ITALIAN TRANSLATION**	
Prestarle piena attenzione	
**BACKWARD TRANSLATION**	
Giving you full attention	
**FINAL ITALIAN VERSION**	
Prestarle piena attenzione	

ITEM # 8

ORIGINAL ENGLISH WORDING	COMMENTS/QUESTIONS
Talking with you about your feelings concerning the possibility that you might get sicker	
**ITALIAN TRANSLATION 1**	
Parlare con lei dei suoi sentimenti riguardo alla possibilità di aggravarsi	
**ITALIAN TRANSLATION 2**	
Parlare con lei di come si sente riguardo alla possibilità che lei possa peggiorare	
**RECONCILED ITALIAN TRANSLATION**	
Parlare con lei dei suoi pensieri riguardo alla possibilità di peggiorare	

Translation report QOC from US English to Italian

**BACKWARD TRANSLATION**	Not sure if "suoi" refers to the doctor's or the patient's thoughts
Discussing your thoughts on the chances of your condition worsening	
**PRE FINAL ITALIAN VERSION**	'Chance' (singular) = 'possibility'<br>'Eventualità'?<br>'Eventualità' seems a smoother, lighter term than 'possibilità', but might not be not equivalent to the original 'possibility'<br><br>*I believe that Item #8 ('talking about the patient's feelings – emotions') is not clear that this is asking about the emotional component of the discussion "Discussing your thoughts on the chances of your condition worsening" and I wonder if the translation of "sentimenti" (similar to English 'sentiments') would not be the better choice here.*
Parlare con lei dei suoi pensieri riguardo alla possibilità di peggiorare	
**FINAL ITALIAN VERSION**	The Panel discussed this point, and agreed that 'pensieri' is conceptually equivalent to 'feelings' here
Parlare con lei dei suoi pensieri riguardo alla possibilità di peggiorare	

ITEM # 9

ORIGINAL ENGLISH WORDING	COMMENTS/QUESTIONS
Talking to you about the details concerning the possibility that you might get sicker	
**ITALIAN TRANSLATION 1**	
Parlare con lei di tutti i dettagli relativi alla possibilità di un aggravamento della malattia	
**ITALIAN TRANSLATION 2**	
Parlare con lei dei dettagli che riguardano la possibilità che lei possa peggiorare	
**RECONCILED ITALIAN TRANSLATION**	
Parlare con lei di tutti i dettagli nell'ipotesi di un peggioramento della malattia	
**BACKWARD TRANSLATION**	
Discussing the details of what might happen if your condition worsened	
**PRE FINAL ITALIAN VERSION**	The focus is on the chance to worsen or on the new (worsened) status?
Parlare con lei di tutti i dettagli relativi alla possibilità di peggiorare	

	'Details' may concern the possibility to worsen, or the worsened disease…<br>Is it possible that item # 8 concerns the competence of the doc to share patient's concerns about the possibility to worsen, and item # 9 the competence of the doc to provide detailed infoon what this worsening could be like? See e.g. the OPTION scaleor other similar scales<br>This seems to be the case!<br>*I believe the that Item #9 ('[...] talking about details – information') about getting sicker is acceptable as you have it here "Discussing the details of what might happen if your condition worsened".*
**FINAL ITALIAN VERSION**	
Parlare con lei di tutti i dettagli relativi alla possibilità di peggiorare	

ITEM # 10

ORIGINAL ENGLISH WORDING	COMMENTS/QUESTIONS
Talking to you about how long you might have to live	
**ITALIAN TRANSLATION 1**	
Parlare con lei di quale possa essere la sua aspettativa di vita	
**ITALIAN TRANSLATION 2**	
Parlare con lei di quanto tempo le possa restare da vivere	
**RECONCILED ITALIAN TRANSLATION**	'Aspettativa di vita' should be preferred and can be considered a lay term
Parlare con lei di quale possa essere la sua aspettativa di vita	
**BACKWARD TRANSLATION**	
Talking with you about what your life expectancy might be	
**FINAL ITALIAN VERSION**	
Parlare con lei di quale possa essere la sua aspettativa di vita	

Translation report QOC from US English to Italian

ITEM # 11

ORIGINAL ENGLISH WORDING	COMMENTS/QUESTIONS
Talking to you about what dying might be like	
**ITALIAN TRANSLATION 1**	
Parlare con lei di come potrebbe essere la morte	
**ITALIAN TRANSLATION 2**	
Parlare con lei di come possa essere morire	
**RECONCILED ITALIAN TRANSLATION**	
Parlare con lei di come potrebbe essere il morire	
**BACKWARD TRANSLATION**	
Talking with you about what dying might be like	
**FINAL ITALIAN VERSION**	
Parlare con lei di come potrebbe essere il morire	

ITEM # 12

ORIGINAL ENGLISH WORDING	COMMENTS/QUESTIONS
Talking with your loved ones about what your dying might be like	
**ITALIAN TRANSLATION 1**	
Parlare con i suoi cari di come potrebbe essere la sua morte	
**ITALIAN TRANSLATION 2**	
Parlare con i suoi cari di come possa essere la sua morte	
**RECONCILED ITALIAN TRANSLATION**	
Parlare con i suoi cari di come potrebbe essere la sua morte	
**BACKWARD TRANSLATION**	
Talking with your loved ones about what your dying might be like	
**FINAL ITALIAN VERSION**	
Parlare con i suoi cari di come potrebbe essere la sua morte	

ITEM # 13

ORIGINAL ENGLISH WORDING	COMMENTS/QUESTIONS
Involving you in the decisions about the treatments that you want if you get too sick to speak for yourself	
**ITALIAN TRANSLATION 1**	
Coinvolgerla nelle decisioni riguardo alle cure che desidera ricevere se si ammala troppo gravemente per potersi esprimere autonomamente	
**ITALIAN TRANSLATION 2**	
Coinvolgerla nelle decisioni sulle terapie che lei vuole nel caso fosse troppo malato per potersi esprimere	
**RECONCILED ITALIAN TRANSLATION**	
Coinvolgerla nelle decisioni riguardo alle cure che desidera ricevere nel caso fosse così malato da non potersi esprimere	
**BACKWARD TRANSLATION**	
Involving you in decisions on what medical care you wish to receive if you are too sick to speak for yourself	
**PRE FINAL ITALIAN VERSION**	See QUERY 4 above
Coinvolgerla nelle decisioni riguardo alle cure che desidera ricevere nel caso fosse così malato da non potersi esprimere	
**FINAL ITALIAN VERSION**	
Coinvolgerla nelle decisioni riguardo alle terapie che desidera ricevere nel caso fosse così malato da non potersi esprimere	

ITEM # 14

ORIGINAL ENGLISH WORDING	COMMENTS/QUESTIONS
Asking about the things in life that are important to you	
**ITALIAN TRANSLATION 1**	
Chiederle quali siano per lei le cose importanti della vita	

ITALIAN TRANSLATION 2	
Chiedere quali sono le cose della vita importanti per lei	
RECONCILED ITALIAN TRANSLATION	
Chiederle quali sono per lei le cose importanti della vita	
BACKWARD TRANSLATION	
Asking about the things in life that are important to you	
FINAL ITALIAN VERSION	
Chiederle quali sono per lei le cose importanti della vita	

ITEM # 15

ORIGINAL ENGLISH WORDING	COMMENTS/QUESTIONS
Respecting the things in your life that are important to you	
ITALIAN TRANSLATION 1	
Rispettare le cose della sua vita che per lei sono importanti	
ITALIAN TRANSLATION 2	
Rispettare le cose della sua vita che sono importanti per lei	
RECONCILED ITALIAN TRANSLATION	
Rispettare le cose della sua vita che sono importanti per lei	
BACKWARD TRANSLATION	
Respecting the things in life that are important to you	
FINAL ITALIAN VERSION	
Rispettare le cose della sua vita che sono importanti per lei	

ITEM # 16

ORIGINAL ENGLISH WORDING	COMMENTS/QUESTIONS
Asking about your spiritual or religious beliefs	
ITALIAN TRANSLATION 1	
Chiederle quali siano le sue convinzioni spirituali o religiose	
ITALIAN TRANSLATION 2	

Farle domande sulla sua fede spirituale o religione	
RECONCILED ITALIAN TRANSLATION	
Chiederle quali siano i suoi valori spirituali o religiosi	
BACKWARD TRANSLATION	Beliefs vs values: In italian ‚beliefs‘ is ‚credenze‘ which has a devaluative meaning<br>The Panel agreed on ‘valori‘
Asking about your spiritual or religious values	
FINAL ITALIAN VERSION	
Chiederle quali siano i suoi valori spirituali o religiosi	

ITEM # 17

ORIGINAL ENGLISH WORDING	COMMENTS/QUESTIONS
Respecting your spiritual or religious beliefs	
ITALIAN TRANSLATION 1	
Rispettare le sue convinzioni spirituali o religiose	
ITALIAN TRANSLATION 2	
Rispettare la sua fede spirituale o religione	
RECONCILED ITALIAN TRANSLATION	
Rispettare i suoi valori spirituali o religiosi	
BACKWARD TRANSLATION	
Respecting your spiritual or religious values	
FINAL ITALIAN VERSION	
Rispettare i suoi valori spirituali o religiosi	

INTRODUCTION TO ITEM 18 AND 19

ORIGINAL ENGLISH WORDING	COMMENTS/QUESTIONS
The next two questions ask you to rate how comfortable this doctor is in talking about dying and about how well this doctor communicates with you	
ITALIAN TRANSLATION 1	I found a lot difficult the translation of "comfortable", which reflects an easiness to communicate from both parties. A sort of two-way meaning, while in Italian ‘a suo agio’ only reflects
Nelle prossime due domande le chiediamo di esprimere una valutazione su quanto il medico sia disponibile e sereno nel parlare della morte, e quanto bene comunichi con	

lei.	one meaning (here how the doctor feels), not the other (his openness to communication/discussion)
**ITALIAN TRANSLATION 2**	
Le prossime due domande le chiedono di valutare quanto questo medico si senta a proprio agio nel parlare di morire e di quanto bene questo medico comunichi con lei	
**RECONCILED ITALIAN TRANSLATION**	To be discussed within the Panel how to translate at best 'confortable'.<br>First version: Le prossime due domande le chiedono di valutare quanto il medico riesca a parlare in modo attento/adeguato, /sensibile e /coinvolto/disponibile/con naturalezza) della morte, e quanto bene comunichi con lei.<br>The Panel agreed on: quanto il medico è a suo agio
Le prossime due domande le chiedono di valutare quanto il medico è a suo agio nel parlare del morire e quanto bene comunichi con lei.	
**BACKWARD TRANSLATION**	
The next two questions are about how comfortable you think your doctor is talking with you about dying and how well he/she communicates with you.	
**PRE FINAL ITALIAN VERSION**	In Italian 'sia' instead of 'e': Agreed
Le prossime due domande le chiedono di valutare quanto il medico sia a suo agio nel parlare del morire e quanto bene comunichi con lei.	
**FINAL ITALIAN VERSION**	'Il suo medico' to increase consistency (see page 5)
Le prossime due domande le chiedono di valutare quanto il suo medico sia a suo agio nel parlare del morire e quanto bene comunichi con lei.	

ITEM # 18

ORIGINAL ENGLISH WORDING	COMMENTS/QUESTIONS
How comfortable do you feel your doctor is talking about dying?	
**ITALIAN TRANSLATION 1**	
Quanto le sembra disponibile e sereno il suo medico nel parlare della morte?	
**ITALIAN TRANSLATION 2**	
Quanto sente che il suo medico è a proprio agio nel parlare di morire?	
**RECONCILED ITALIAN TRANSLATION**	Same as before ('comfortable')

Quanto le sembra a proprio agio il suo medico nel parlare del morire?	
**BACKWARD TRANSLATION**	
How comfortable does your doctor seem to be talking about dying?	
**FINAL ITALIAN VERSION**	
Quanto ritiene che il suo medico sia a proprio agio nel parlare del morire?	

INSTRUCTION ITEM 18

ORIGINAL ENGLISH WORDING	COMMENTS/QUESTIONS
On the scale below, 0 = "not at all comfortable" and 10 = "extremely comfortable" (Circle one number)	
**ITALIAN TRANSLATION 1**	
Sulla scala che segue, 0 = "estremamente a disagio" e 10 = "estremamente disponibile e sereno" (Cerchiare un numero)	
**ITALIAN TRANSLATION 2**	
Nella scala sotto, 0 = "per nulla a proprio agio" e 10 = "decisamente proprio agio (Cerchiare un numero)	
**RECONCILED ITALIAN TRANSLATION**	Same as before ('comfortable')
Sulla scala che segue, dove "0" = per nulla a proprio agio e "10" = del tutto a proprio agio, segni il numero che corrisponde alla sua valutazione	
**BACKWARD TRANSLATION**	NOT TO BE CONSIDERED: The back translator received a wrong phrase to translate (error in the procedure)
Please answer using the following scale from "0" = not at all comfortable to "10" = best imaginable	
**PRE FINAL ITALIAN VERSION**	
Sulla scala che segue, dove "0" = per nulla a proprio agio e "10" = del tutto a proprio agio, segni il numero che corrisponde alla sua valutazione	
**FINAL ITALIAN VERSION**	
Sulla scala che segue, dove "0" = per nulla a proprio agio e "10" = del tutto a proprio agio, **[segni]/[selezioni]** il numero che corrisponde alla sua valutazione	Depending on the format (paper or electronic, see page 5)

Translation report QOC from US English to Italian

ITEM # 19

ORIGINAL ENGLISH WORDING	COMMENTS/QUESTIONS
Overall, how would you rate this doctor's communication with you?	
**ITALIAN TRANSLATION 1**	
Nel complesso, come valuta il modo in cui il suo medico comunica con lei?	
**ITALIAN TRANSLATION 2**	
Complessivamente, come valuta la comunicazione di questo medico con lei?	
**RECONCILED ITALIAN TRANSLATION**	
Complessivamente, come valuta il modo in cui questo medico comunica con lei?	
**BACKWARD TRANSLATION**	
Overall, how do you rate how this doctor communicates with you?	
**PRE FINAL ITALIAN VERSION**	
Complessivamente, come valuta il modo in cui questo medico comunica con lei?	
**FINAL ITALIAN VERSION**	'Il suo medico' to increase consistency (see page 5)
Complessivamente, come valuta il modo in cui il suo medico comunica con lei?	

INSTRUCTION ITEM 19

ORIGINAL ENGLISH WORDING	COMMENTS/QUESTIONS
On the scale below, 0 = "the very worst I could imagine" and 10 = "the very best I could imagine" (Circle one number)	
**ITALIAN TRANSLATION 1**	
Sulla scala che segue, 0 = "nel modo migliore che posso immaginare" e 10 = "nel modo peggiore che posso immaginare" (Cerchiare un numero)	
**ITALIAN TRANSLATION 2**	
Nella scala sotto, 0= "decisamente peggio di quanto potessi immaginare" e 10 = "decisamente meglio di quanto potessi immaginare" (Cerchiare un numero)	

Translation report QOC from US English to Italian

RECONCILED ITALIAN TRANSLATION	
Sulla scala che segue, dove "0" rappresenta il peggio che possa immaginare e “10” il meglio che possa immaginare, segni il numero che corrisponde alla sua valutazione.	
**BACKWARD TRANSLATION**	
Please answer using the following scale from “0” = Worst imaginable to “10” = best imaginable	
**PRE FINAL ITALIAN VERSION**	
Sulla scala che segue, dove "0" rappresenta il peggio che possa immaginare e “10” il meglio che possa immaginare, segni il numero che corrisponde alla sua valutazione.	
**FINAL ITALIAN VERSION**	
Sulla scala che segue, dove "0" rappresenta il peggio che possa immaginare e “10” il meglio che possa immaginare, **[segni]/[selezioni** il numero che corrisponde alla sua valutazione.	Depending on the format (paper or electronic, see page 5)
