# Supplementary material for: Italian cross-cultural adaptation of the Quality of Communication questionnaire and the 4-item advance care planning engagement questionnaire
Source: PLoS One. 2023 Mar 23;18(3):e0282960. doi: 10.1371/journal.pone.0282960 (PMC10035811; doi:10.1371/journal.pone.0282960)
Supplement: S2 File — (PDF) [file pone.0282960.s002.pdf]

Translation Report 4-item ACP Engagement Survey (patient self-assessed and interviewer-administered versions) from English to Italian

S2 File. Italian 4-ACP Engagement translation grid

PATIENT SELF-ADMINISTERED VERSION

COMMENTS/QUESTIONS MADE BY:		
PP (Consultant Italian translator 1)	AS (Researcher 1)	AG (Researcher 3)
FB (Consultant Italian translator 2)	SV (Researcher 2)	PK (expert patient)
LR (Consultant backward translator)	MAF (Lay person)	LDP (bioethicist, consulted after the reconciled Italian version)
		RL Sudore (ACP-E Author), L Lowe and other members of the ACP-E team

TITLE

ORIGINAL ENGLISH WORDING	COMMENTS/QUESTIONS
**4-item ACP Engagement Survey, Participant Version**	
**ITALIAN TRANSLATION 1**	
Questionario a 4 item sul percorso PAC, versione autocompilata	
**ITALIAN TRANSLATION 2**	
Questionario ACP in 4 parti sul coinvolgimento attivo, Versione del partecipante	
**RECONCILED ITALIAN TRANSLATION**	I would go with"autocompilata DAL paziente" or just "versione paziente"<br>I vote"versione paziente"
Questionario sulla partecipazione attiva alla Pianificazione Condivisa delle Cure, versione a 4-item autocompilata per il paziente	
**BACKWARD TRANSLATION**	
Advance Care Planning Active Engagement Questionnaire, patient self-administered 4-item version	
**PRE FINAL ITALIAN VERSION**	As for other patient reported outcome measures, we'll keep the original (US English) title and add the Italian translation as a sub-title.
Questionario sulla partecipazione attiva del paziente alla Pianificazione Condivisa delle Cure, versione a 4-item autocompilata	
**FINAL ITALIAN VERSION**	
Questionario sulla partecipazione attiva del paziente alla Pianificazione Condivisa delle Cure, modulo a 4 domande, versione autocompilata	

Translation Report 4-item ACP Engagement Survey (patient self-assessed and interviewer-administered versions) from English to Italian

SUB-TITLE

ORIGINAL ENGLISH WORDING	COMMENTS/QUESTIONS
PREPARATION ENGAGEMENT	
**ITALIAN TRANSLATION 1**	"Engagement", difficult to render in Italian
PERCORSO DI PREPARAZIONE	
**ITALIAN TRANSLATION 2**	
COINVOLGIMENTO ATTIVO NELLA PREPARAZIONE	
**RECONCILED ITALIAN TRANSLATION**	Preparation to participation???<br>Not discussed whether to leave "questionario sul"<br>I would leave it
Questionario sul percorso di partecipazione	
**BACKWARD TRANSLATION**	
Questionnaire on the Engagement Process	
**PRE FINAL ITALIAN VERSION**	'Engagement Preparation' instead?
Preparazione e predisposizione	
**FINAL ITALIAN VERSION**	
Preparazione e predisposizione alla Pianificazione Condivisa delle Cure	

INTRODUCTION *(first statement)*

ORIGINAL ENGLISH WORDING	COMMENTS/QUESTIONS
We will now ask you about your experiences and opinions.	
**ITALIAN TRANSLATION 1**	This seems to be taken from the interviewer-administered version ("Now" is for the inteviewer who reads out)
Le chiediamo di rispondere ad alcune domande circa le sue esperienze ed opinioni.	
**ITALIAN TRANSLATION 2**	
Adesso le faremo domande in merito alle sue esperienze e opinioni	
**RECONCILED ITALIAN TRANSLATION**	
Le domande seguenti riguarderanno le sue esperienze ed opinioni	

Translation Report 4-item ACP Engagement Survey (patient self-assessed and interviewer-administered versions) from English to Italian

**BACKWARD TRANSLATION**	
The following questions are about your experiences and opinions	
**FINAL ITALIAN TRANSLATION**	
Le domande seguenti riguardano le sue esperienze e opinioni	

INTRODUCTION *(second statement)*

**ORIGINAL ENGLISH WORDING**	**COMMENTS/QUESTIONS**
We may ask you about things that you have already done, or have not thought about at all.	
**ITALIAN TRANSLATION 1**	
Le domande potranno riguardare cose che forse ha già fatto, o a cui magari non ha neppure pensato.	
**ITALIAN TRANSLATION 2**	
Le potremmo fare domande in merito a cose che lei ha già fatto o che non ha per nulla considerato.	
**RECONCILED ITALIAN TRANSLATION**	'Cose' is a bit colloquial/diminishing. 'Aspetti'? 'Argomenti'?
Le domande potranno riguardare cose che forse ha già fatto, o che non ha neppure preso in considerazione	
**BACKWARD TRANSLATION**	
The questions may be about things you have already done or might never have considered.	
**FINAL ITALIAN VERSION**	
Potranno riferirsi ad aspetti che forse ha già affrontato, o che non ha neppure preso in considerazione	

INTRODUCTION *(third line)*

**ORIGINAL ENGLISH WORDING**	**COMMENTS/QUESTIONS**
Please try to answer as honestly as you can.	
**ITALIAN TRANSLATION 1**	
Cerchi di rispondere con la massima sincerità possibile.	
**ITALIAN TRANSLATION 2**	

Per favore, provi a rispondere il più onestamente possibile.	
**RECONCILED ITALIAN TRANSLATION**	
Risponda nel modo più sincero possibile	
**BACKWARD TRANSLATION**	
Please reply as honestly as you can.	
**PRE FINAL ITALIAN VERSION**	**QUERY # 1:** The 4-item version has 4 items and a number of sub-titles, headings and repetitions (e,g, the current sentence). This can be a challenge to the participant. A proposal to slightly revise this 4-item version is prepared on this regard to be discussed with the scale Author (separate document). Specifically, the sentence deleted was replaced with sentence on page 5, which is also more complete. *ANSWER: It is OK to remove the first statement.*
Risponda nel modo più sincero possibile	
**FINAL ITALIAN VERSION**	
~~Risponda nel modo più sincero possibile~~ (REMOVED)	

INTRODUCTION *(4th line)*

**ORIGINAL ENGLISH WORDING**	**COMMENTS/QUESTIONS**
Over the next few sections we will be asking you about 2 topics:	
**ITALIAN TRANSLATION 1**	
Nei paragrafi che seguono toccheremo 2 argomenti:	
**ITALIAN TRANSLATION 2**	
Nelle prossime sezioni le faremo domande in merito a due argomenti:	
**RECONCILED ITALIAN TRANSLATION**	It was: "Nelle prossime sezioni le faremo domande in merito a due argomenti" (before 25/05/2021) I prefer the reconciled Italian version
Nelle prossime sezioni le domande saranno in merito a due argomenti:	
**BACKWARD TRANSLATION**	
In the following sections the questions are on two topics:	
**FINAL ITALIAN VERSION**	

Translation Report 4-item ACP Engagement Survey (patient self-assessed and interviewer-administered versions) from English to Italian

Nelle prossime sezioni le domande saranno in merito a due argomenti:	The panel proposes the following: *'Please give us your honest opinions. There are no right or wrong answers.'* (taken from INTRODUCTION (last sentence) not to be repeated any more (see **QUERY # 1** above).

INTRODUCTION *(topics)*

ORIGINAL ENGLISH WORDING	COMMENTS/QUESTIONS
1. Medical decision makers, or surrogates<br>2. Deciding what matters most in life	
**ITALIAN TRANSLATION 1**	# 2 is has been translated 'che cosa conta di più…' however 'priorities' could be considered more appropriate<br>I suggest "cosa è più importante"
1. Fiduciario per le decisioni mediche, o sostituto<br>2. Decidere che cosa conta di più nella vita	
**ITALIAN TRANSLATION 2**	
1. Il decisore medico o il decisore medico sostitutivo<br>2. Decidere cosa conta di più nella vita	
**RECONCILED ITALIAN TRANSLATION**	"Fiduciario" is what the Law says and it prevents any confusion<br>Right, we must use 'fiduciario'
1. Il fiduciario<br>2. Decidere cosa conta di più nella vita	
**BACKWARD TRANSLATION**	
1. The surrogate decision-maker<br>2. Deciding what matters most in life	
**PRE FINAL ITALIAN VERSION**	We propose to skip this list as well (see QUERY # 1)
1. Il fiduciario<br>2. Decidere cosa conta di più nella vita	
**FINAL ITALIAN VERSION**	
1. ~~Il fiduciario~~<br>2. ~~Decidere cosa conta di più nella vita~~ (REMOVED)	

Translation Report 4-item ACP Engagement Survey (patient self-assessed and interviewer-administered versions) from English to Italian

INTRODUCTION *(last statement)*

ORIGINAL ENGLISH WORDING	COMMENTS/QUESTIONS
Please give us your honest opinions. There are no right or wrong answers.	
**ITALIAN TRANSLATION 1**	
Esprima con sincerità le sue opinioni. Non ci sono risposte giuste e risposte sbagliate.	
**ITALIAN TRANSLATION 2**	
Per favore, ci dia la sua opinione sincera. Non ci sono risposte giuste o sbagliate.	
**RECONCILED ITALIAN TRANSLATION**	
Esprima con sincerità le sue opinioni. Non ci sono risposte giuste o sbagliate.	
**BACKWARD TRANSLATION**	
Please answer as honestly as you can. There are no right or wrong answers.	
**FINAL ITALIAN VERSION**	This sentence replaces sentence on page 3 to avoid repetitions (it is a 4-item version, no need to repeat this content). See QUERY # 1 above.
Esprima con sincerità le sue opinioni. Non ci sono risposte giuste o sbagliate.	

TOPIC 1. *(title)*

ORIGINAL ENGLISH WORDING	COMMENTS/QUESTIONS
1. Medical Decision Makers	
**ITALIAN TRANSLATION 1**	
1. Fiduciario per le decisioni mediche	
**ITALIAN TRANSLATION 2**	
1. Decisori medici	
**RECONCILED ITALIAN TRANSLATION**	A legal person with a hierarchical order, so singular is fine
1. Il fiduciario	
**BACKWARD TRANSLATION**	
1. The surrogate decision-maker	
**PRE FINAL ITALIAN VERSION**	The number 1. could be skipped (see QUERY # 1)
1. Il fiduciario	
**FINAL ITALIAN VERSION**	
Il fiduciario	

TOPIC 1. *(introduction)*

ORIGINAL ENGLISH WORDING	COMMENTS/QUESTIONS
This question asks about medical decision makers, or a family member or friend who can make decisions for you if you were to become too sick to make your own decisions.	
**ITALIAN TRANSLATION 1**	
Questa domanda riguarda il fiduciario per le decisioni mediche, ad esempio un suo parente o amico che possa prendere decisioni a suo nome nel momento in cui lei fosse troppo malato per decidere autonomamente.	
**ITALIAN TRANSLATION 2**	
Questa domanda è sui decisori medici, o un membro della famiglia oppure un amico che può prendere decisioni al suo posto nel caso lei diventasse troppo malato per prendere decisioni da solo.	
**RECONCILED ITALIAN TRANSLATION**	Modified on 25/05 after exchange with PK (who left the first meeting for another commitment) on the interviewer version. Fine with me
Questa domanda riguarda il fiduciario, ad esempio un membro della famiglia o un amico, che possa prendere decisioni a suo nome nel caso lei non fosse in grado di farlo	
**BACKWARD TRANSLATION**	
This question is about your surrogate decision-maker, who may, for example, be a family member or friend who can make decisions on your behalf if you are too sick.	
**FINAL ITALIAN VERSION**	
Questa domanda riguarda il fiduciario, ad esempio un membro della famiglia o un amico, che possa prendere decisioni a suo nome nel caso lei non fosse in grado di farlo	

Topic 1. *(question 1)*

ORIGINAL ENGLISH WORDING	COMMENTS/QUESTIONS
How ready are you to sign OFFICIAL PAPERS naming a person or group of people to make medical decisions for you?	
**ITALIAN TRANSLATION 1**	To comply with the Italian Law, 'group' is left out

Quanto si sente pronto a firmare un DOCUMENTO UFFICIALE in cui nomina una o più persone incaricate di prendere decisioni mediche a nome suo?	
**ITALIAN TRANSLATION 2**	
Lei quanto è pronto a firmare dei DOCUMENTI UFFICIALI che nominano una persona o un gruppo di persone a prendere decisioni cliniche al suo posto?	
**RECONCILED ITALIAN TRANSLATION**	
Quanto si sente pronto a firmare un DOCUMENTO UFFICIALE in cui nomina una o più persone incaricate di prendere decisioni mediche a suo nome?	
**BACKWARD TRANSLATION**	
How ready do you feel to sign an OFFICIAL DOCUMENT appointing one or more people to make medical decisions on your behalf?	
**FINAL ITALIAN VERSION**	
Quanto si sente pronto a firmare un DOCUMENTO UFFICIALE in cui nomina una o più persone incaricate di prendere decisioni mediche a suo nome?	

Possible reply a)

ORIGINAL ENGLISH WORDING	COMMENTS/QUESTIONS
a. I have never thought about it	
**ITALIAN TRANSLATION 1**	
a. Non ci ho mai pensato	
**ITALIAN TRANSLATION 2**	
a. Non ci ho mai pensato	
**RECONCILED ITALIAN TRANSLATION 1**	
a. Non ci ho mai pensato	
**BACKWARD TRANSLATION**	
a. I have never thought about it	
**PRE FINAL ITALIAN VERSION**	The letter a. could be replaced by a box (see QUERY # 1)
a. Non ci ho mai pensato	
**FINAL ITALIAN VERSION**	
☐ Non ci ho mai pensato	

Possible reply b)

ORIGINAL ENGLISH WORDING	COMMENTS/QUESTIONS
b. I have thought about it, but I am not ready to do it	
**ITALIAN TRANSLATION 1**	
b. Ci ho pensato, ma non mi sento pronto a farlo	
**ITALIAN TRANSLATION 2**	
b. Ci ho pensato ma non sono pronto a farlo	
**RECONCILED ITALIAN TRANSLATION**	
b. Ci ho pensato, ma non mi sento pronto a farlo	
**BACKWARD TRANSLATION**	I am not sure if the Italian means “not ready – i.e. it is too soon” or “not ready – i.e. not willing”.
b. I have thought about it, but I do not feel ready to do it	
**FINAL ITALIAN VERSION**	The letter b. could be replaced by a box (see QUERY # 1)
☐ Ci ho pensato, ma non mi sento pronto a farlo	

Possible reply c)

ORIGINAL ENGLISH WORDING	COMMENTS/QUESTIONS
c. I am thinking about doing it in the next 6 months	
**ITALIAN TRANSLATION 1**	
c. Penso che lo farò nei prossimi 6 mesi	
**ITALIAN TRANSLATION 2**	
c. Sto pensando di farlo nei prossimi 6 mesi	
**RECONCILED ITALIAN TRANSLATION**	
c. Credo che lo farò nei prossimi 6 mesi	Penso che potrei farlo
**BACKWARD TRANSLATION**	
c. I think I will do it in the next 6 months	
**FINAL ITALIAN VERSION**	The letter c. could be replaced by a box (see QUERY # 1)
☐ Penso che potrei farlo nei prossimi 6 mesi	

Possible reply d)

ORIGINAL ENGLISH WORDING	COMMENTS/QUESTIONS
d. I am definitely planning to do it in the next 30 days	
**ITALIAN TRANSLATION 1**	
d. Lo farò certamente nei prossimi 30 giorni	
**ITALIAN TRANSLATION 2**	
d. Ho decisamente in programma di farlo nei prossimi 30 giorni	
**RECONCILED ITALIAN TRANSLATION**	
d. Ho proprio intenzione di farlo nei prossimi 30 giorni	
**BACKWARD TRANSLATION**	
**d.** I definitely will do it in the next 30 days	
**FINAL ITALIAN VERSION**	The letter d. could be replaced by a box (see QUERY # 1)
☐ Ho proprio intenzione di farlo nei prossimi 30 giorni	

Possible reply e)

ORIGINAL ENGLISH WORDING	COMMENTS/QUESTIONS
e. I have already done it	
**ITALIAN TRANSLATION 1**	
e. L'ho già fatto	
**ITALIAN TRANSLATION 2**	
e. L'ho già fatto	
**RECONCILED ITALIAN TRANSLATION**	
e. L'ho già fatto	
**BACKWARD TRANSLATION**	
**e.** I have already done it	
**FINAL ITALIAN VERSION**	The letter e. could be replaced by a box (see QUERY # 1)
☐ L'ho già fatto	

As a result of QUERIES # 3,4 specific for the interviewer version (pages 25-26) the response option 'preferisco non rispondere' was added. This applies to all the 4 items.

TOPIC 2. *(title)*

ORIGINAL ENGLISH WORDING	COMMENTS/QUESTIONS
2. What Matters Most in Life	
**ITALIAN TRANSLATION 1**	
2. Che cosa conta di più nella vita	
**ITALIAN TRANSLATION 2**	
2. Cosa importa di più nella vita	
**RECONCILED ITALIAN TRANSLATION**	
2. Cosa conta di più nella vita	
**BACKWARD TRANSLATION**	
2. What matters most in life	
**PRE FINAL ITALIAN VERSION**	The number 2. could be skipped (see QUERY # 1)
2. Cosa conta di più nella vita	
**FINAL ITALIAN VERSION**	
Cosa conta di più nella vita	

TOPIC 2. *(introduction...)*

ORIGINAL ENGLISH WORDING	COMMENTS/QUESTIONS
The next few questions are about specific medical treatments that people may or may not want if they were very sick or at the end of their life.	
**ITALIAN TRANSLATION 1**	
Le prossime domande riguardano le specifiche cure mediche che lei, come ogni persona, può voler o non voler ricevere se è in condizioni molto gravi o in fin di vita.	
**ITALIAN TRANSLATION 2**	

Le prossime domande riguardano particolari terapie mediche che le persone possono volere o non volere se fossero molto malate o alla fine della loro vita.	
**RECONCILED ITALIAN TRANSLATION 1**	Modified on 25/05 after the interview version<br>Fine with me
Le prossime domande riguardano i trattamenti sanitari che le persone possono volere o non voler ricevere trovandosi in condizioni molto gravi o in fin di vita.	
**BACKWARD TRANSLATION**	
The next questions are about health care treatments that people may or may not want to receive when they are seriously ill or dying.	
**PRE FINAL ITALIAN VERSION**	**Query # 2:** Is there any reason for using 'medical treatments' here and medical care' in TOPIC 2 (questions 2-4)?<br>*ANSWER: No specific reason. It is OK to change "medical treatments" to "medical care" for consistency.*
Le prossime domande riguardano il tipo di trattamenti sanitari che le persone possono volere o non voler ricevere trovandosi in condizioni molto gravi o in fin di vita.	
**FINAL ITALIAN VERSION**	
Le prossime domande riguardano il tipo di assistenza e trattamenti sanitari che le persone possono volere o non voler ricevere trovandosi in condizioni molto gravi o in fin di vita.	

TOPIC 2. *(…introduction)*

**ORIGINAL ENGLISH WORDING**	**COMMENTS/QUESTIONS**
For instance, some people know they would want to be on a breathing machine. Other people know they would never want to be on a breathing machine.	
**ITALIAN TRANSLATION 1**	
Ad esempio, alcune persone sanno che vorrebbero essere collegate ad un respiratore. Altre persone sanno che non vorrebbero mai essere collegate ad un respiratore.	
**ITALIAN TRANSLATION 2**	
Per esempio, alcune persone sanno che vorrebbero essere attaccate ad un respiratore. Altre persone sanno che non vorrebbero mai essere attaccate ad un respiratore.	
**RECONCILED ITALIAN TRANSLATION**	

Per esempio, alcune persone sanno che accetterebbero di essere tenute in vita con un respiratore. Altre persone sanno che non lo vorrebbero mai.	
**BACKWARD TRANSLATION**	
For instance, some people know they would agree to be kept alive on a ventilator. Other people know they would never want that.	
**FINAL ITALIAN VERSION**	'Mantenuto in vita' (kept alive) helps clarifying the meaning of this sentence in Italian. The back translation contains this specification, without changing the conceptual equivalence
Per esempio, alcune persone sanno che accetterebbero di essere tenute in vita con un respiratore. Altre persone sanno che non lo vorrebbero mai.	

[The last line of introduction is the same as at page 4, "Introduction, last statement"]

Topic 2. *(question 2)*

ORIGINAL ENGLISH WORDING	COMMENTS/QUESTIONS
2. How ready are you to talk to your MEDICAL DECISION MAKER about the kind of medical care you would want if you were very sick or near the end of life?	
**ITALIAN TRANSLATION 1**	
2. Quanto si sente pronto a parlare al suo FIDUCIARIO PER LE DECISIONI MEDICHE del tipo di assistenza medica che vorrebbe ricevere se fosse in condizioni molto gravi o in fin di vita?	
**ITALIAN TRANSLATION 2**	
2. Quanto è pronto a parlare con il suo DECISORE MEDICO riguardo al tipo di assistenza medica che lei vorrebbe se fosse molto malato o prossimo alla fine della vita ?	
**RECONCILED ITALIAN TRANSLATION**	
Quanto si sente pronto a parlare con il suo FIDUCIARIO del tipo di trattamenti sanitari che vorrebbe ricevere se fosse in condizioni molto gravi o in fin di vita?	
**BACKWARD TRANSLATION**	
How ready do you feel to talk with your SURROGATE DECISION-MAKER about the type of care and medical treatment you would want to receive if you were seriously ill or dying?	
**FINAL ITALIAN VERSION**	See **Query # 2** above
2. Quanto si sente pronto a parlare con il suo FIDUCIARIO del tipo di assistenza e trattamenti sanitari che vorrebbe ricevere se fosse in condizioni molto gravi o in fin di vita?	

Topic 2. *(question 3)*

ORIGINAL ENGLISH WORDING	COMMENTS/QUESTIONS
3. How ready are you to talk to your DOCTOR about the kind of medical care you would want if you were very sick or near the end of life?	
**ITALIAN TRANSLATION 1**	
3. Quanto si sente pronto a parlare al suo MEDICO del tipo di assistenza medica che vorrebbe ricevere se fosse in condizioni molto gravi o in fin di vita?	
**ITALIAN TRANSLATION 2**	
3. Quanto è pronto a parlare con il suo MEDICO CURANTE sul tipo di assistenza medica che lei vorrebbe se fosse molto malato o prossimo alla fine della vita ?	
**RECONCILED ITALIAN TRANSLATION**	
3. Quanto si sente pronto a parlare con il suo MEDICO del tipo di trattamenti sanitari che vorrebbe ricevere se fosse in condizioni molto gravi o in fin di vita?	
**BACKWARD TRANSLATION**	
3. How ready do you feel to talk with your DOCTOR about the type of care and medical treatment you would want to receive if you were seriously ill or dying?	
**FINAL ITALIAN VERSION**	
3. Quanto si sente pronto a parlare con il suo MEDICO del tipo di assistenza e trattamenti sanitari che vorrebbe ricevere se fosse in condizioni molto gravi o in fin di vita?	

Topic 2. *(question 4)*

ORIGINAL ENGLISH WORDING	COMMENTS/QUESTIONS
4. How ready are you to SIGN OFFICIAL PAPERS putting your wishes in writing about the kind of medical care you would want if you were very sick or near the end of life?	
**ITALIAN TRANSLATION 1**	
4. Quanto si sente pronto a firmare un DOCUMENTO UFFICIALE che metta per iscritto il tipo di assistenza medica che vorrebbe ricevere se fosse in condizioni molto gravi o in fin di vita?	
**ITALIAN TRANSLATION 2**	

4. Quanto è pronto a FIRMARE DOCUMENTI UFFICIALI mettendo per iscritto le sue volontà in merito al tipo di assistenza medica che lei vorrebbe se fosse molto malato o prossimo alla fine della vita ?	
**RECONCILED ITALIAN TRANSLATION**	
3. Quanto si sente pronto a firmare un DOCUMENTO UFFICIALE che metta per iscritto il tipo di trattamenti sanitari che vorrebbe ricevere se fosse in condizioni molto gravi o in fin di vita?	
**BACKWARD TRANSLATION**	
4. How ready do you feel to sign an OFFICIAL DOCUMENT that puts into writing the type of care and medical treatment you would want to receive if you were seriously ill or dying?	
**FINAL ITALIAN VERSION**	See **Query # 2** above
4. Quanto si sente pronto a firmare un DOCUMENTO UFFICIALE che metta per iscritto il tipo di assistenza e trattamenti sanitari che vorrebbe ricevere se fosse in condizioni molto gravi o in fin di vita?	

For topic 2, question 2 and 4, "Replies from a) to e)" are the same as for Topic 1, question 1.

Possible reply c) (only for Topic 2, Question 3)

ORIGINAL ENGLISH WORDING	COMMENTS/QUESTIONS
c. I am thinking about doing it over the next few visits	
**ITALIAN TRANSLATION 1**	
c. Penso di farlo nel corso delle prossime visite	
**ITALIAN TRANSLATION 2**	
c. Sto pensando di farlo nelle le prossime visite	
**RECONCILED ITALIAN TRANSLATION**	
c. Credo che lo farò in una delle prossime visite	
**BACKWARD TRANSLATION**	
c. I think I will do it at one of my next appointments	
**FINAL ITALIAN VERSION**	The letter c. could be replaced by a box (see QUERY # 1)
☐ Penso che potrei farlo in una delle prossime visite	

Possible reply d) (only for Topic 2, Question 3)

ORIGINAL ENGLISH WORDING	COMMENTS/QUESTIONS
d. I am definitely planning to do it at the next visit	
**ITALIAN TRANSLATION 1**	
d. Lo farò certamente alla prossima visita	
**ITALIAN TRANSLATION 2**	
d. Ho decisamente in programma di farlo alla prossima visita	
**RECONCILED ITALIAN TRANSLATION**	
d. Ho proprio intenzione di farlo alla prossima visita	
**BACKWARD TRANSLATION**	
d. I definitely will do it at my next appointment	
**FINAL ITALIAN VERSION**	The letter d. could be replaced by a box (see QUERY # 1)
☐ Ho proprio intenzione di farlo alla prossima visita	

Addendum: Additional reply options (all 4 items) specific for the "4-item ACP Engagement Survey, Interviewer Version" from English to Italian

Possible reply f)

ORIGINAL ENGLISH WORDING	COMMENTS/QUESTIONS
Not sure	
**ITALIAN TRANSLATION 1**	
Non è sicuro	
**ITALIAN TRANSLATION 2**	
Non (ne sono) sicuro	
**RECONCILED ITALIAN TRANSLATION**	
Non sa (cosa rispondere)	
**BACKWARD TRANSLATION**	
Not sure (what to answer)	
**FINAL ITALIAN VERSION**	**QUERY # 3**: This response option could be present also in the self-assessed version ? ('Non saprei') *ANSWER: These options were included in the original. Please refer to page 6: ACP Engagement Survey. In analysis, these options are considered missing data. You can choose to include or exclude these options.*
Non sa (cosa rispondere)	

Possible reply g)

ORIGINAL ENGLISH WORDING	COMMENTS/QUESTIONS
Refused	
**ITALIAN TRANSLATION 1**	
Rifiuta di rispondere	
**ITALIAN TRANSLATION 2**	

(Mi sono) rifiutato	
**RECONCILED ITALIAN TRANSLATION**	
Rifiuta di rispondere	
**BACKWARD TRANSLATION**	
Refuses to answer	
**FINAL ITALIAN VERSION**	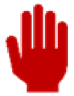 **QUERY # 4**: This response option could be present also in the self-assessed version ? ('Preferisco non rispondere') *ANSWER: These options were included in the original. Please refer to page 6: ACP Engagement Survey. In analysis, these options are considered missing data. You can choose to include or exclude these options.*
Rifiuta di rispondere	

Additional question if reply e) was chosen

ORIGINAL ENGLISH WORDING	COMMENTS/QUESTIONS
OPTIONAL: If they answered, "I have already done it," then ask "When did you do this?"	
**ITALIAN TRANSLATION 1**	
OPZIONALE: Se la persona ha risposto "L'ho già fatto", chiedere "Quando l'ha fatto?"	
**ITALIAN TRANSLATION 2**	
ALTERNATIVA: se rispondono " L'ho già fatto" , allora domandare " Quando lo ha fatto?"	
**RECONCILED ITALIAN TRANSLATION**	
OPZIONALE: Se risponde "L'ho già fatto", chiedere "Quando l'ha fatto?"	
**BACKWARD TRANSLATION**	
OPTIONAL: If the reply is "I have already done it", ask "When did you do it?"	
**FINAL ITALIAN VERSION**	
OPZIONALE: Se risponde "L'ho già fatto", chiedere "Quando l'ha fatto?"	

Possible reply a) to OPTIONAL

ORIGINAL ENGLISH WORDING	COMMENTS/QUESTIONS
Less than 6 months ago	
**ITALIAN TRANSLATION 1**	
Meno di sei mesi fa	
**ITALIAN TRANSLATION 2**	
Meno di 6 mesi fa	
**RECONCILED ITALIAN TRANSLATION**	
Meno di 6 mesi fa	
**BACKWARD TRANSLATION**	
Less than 6 months ago	
**FINAL ITALIAN VERSION**	As it is an interviewer version, it is acceptable to use symbols as for the original version.
≤ 6 mesi fa	

Possible reply b) to OPTIONAL

ORIGINAL ENGLISH WORDING	COMMENTS/QUESTIONS
> 6 months ago	
**ITALIAN TRANSLATION 1**	
Più di sei mesi fa	
**ITALIAN TRANSLATION 2**	
Più di 6 mesi fa	
**RECONCILED ITALIAN TRANSLATION**	
Più di 6 mesi fa	
**BACKWARD TRANSLATION**	
More than 6 months ago	
**FINAL ITALIAN VERSION**	
Più di 6 mesi fa	

Possible reply c) to OPTIONAL

ORIGINAL ENGLISH WORDING	COMMENTS/QUESTIONS
NA	
**ITALIAN TRANSLATION 1**	
NA	
**ITALIAN TRANSLATION 2**	
Non applicabile	
**RECONCILED ITALIAN TRANSLATION**	
NA	
**BACKWARD TRANSLATION**	
N/A	
**FINAL ITALIAN VERSION**	**QUERY # 5**: What does this response option mean ?<br>Please clarify the meaning of NA<br>[No response provided on this query]
NA	

Translation Report 4-item ACP Engagement Survey (patient self-assessed and interviewer-administered versions) from English to Italian

INTERVIEWER-ADMINISTERED VERSION

IMPORTANT NOTICE: Only contents specific for the interviewer-administered version are reported here. The other contents are the same of the self-assessed version, and are in the corresponding translation report

COMMENTS/QUESTIONS MADE BY:			
PP (Consultant Italian translator 1)<br>FB (Consultant Italian translator 2)<br>LR (Consultant backward translator)	AS (Researcher 1)<br>MAF (Lay person)<br>*R Sudore (scale author)*	SV (Researcher2)<br>PK (Expert patient)	AG (Researcher 3)

INTRODUCTION *(first part)*

ORIGINAL ENGLISH WORDING	COMMENTS/QUESTIONS
We will ask about your experiences and opinions. We may ask about things that you have already done, or have not thought about at all. Just answer as honestly as you can	
**ITALIAN TRANSLATION 1**	
Le faremo alcune domande circa le sue esperienze ed opinioni. Potranno riguardare cose che forse ha già fatto, o a cui magari non ha neppure pensato. Cerchi semplicemente di rispondere nel modo più sincero possibile.	
**ITALIAN TRANSLATION 2**	
Le faremo domande in merito alle sue esperienze e opinioni. Le potremo domandare di cose che lei ha già' fatto o che non ha per nulla considerato (di fare). Risponda solo il più onestamente possibile	
**RECONCILED ITALIAN TRANSLATION**	
Le faremo delle domande in merito alle sue esperienze ed opinioni. Le domande potranno riguardare cose che forse ha già fatto, o che non ha neppure preso in considerazione. Risponda nel modo più sincero possibile.	
**BACKWARD TRANSLATION**	

We will be asking you some questions about your experiences and opinions. The questions may be about things you have already done or might never have considered. Please reply as honestly as you can.	
**PRE FINAL ITALIAN VERSION**	After re-assembling the questionnaire we noticed that in Italian the plural is not used for in person interview. For this reason 'Le faremo' (we will be asking) has been replaced with 'Le farò' (I will be asking). Moreover, in line with what we have done in the self-reported version, we added a short sentence explaining the multiple-option choice procedure. The sentece about being sincere has been removed (it is repeated immediately after, see next session).
Le faremo delle domande in merito alle sue esperienze ed opinioni. Le domande potranno riguardare aspetti che forse ha già affrontato, o che non ha neppure preso in considerazione. Risponda nel modo più sincero possibile.	
**FINAL ITALIAN VERSION**	
Le farò delle domande in merito alle sue esperienze ed opinioni. Le domande potranno riguardare aspetti che forse ha già affrontato, o che non ha neppure preso in considerazione. Per ciascuna domanda le proporrò alcune opzioni di risposta tra cui scegliere quella che meglio descrive la sua attuale situazione.	

ORIGINAL ENGLISH WORDING	COMMENTS/QUESTIONS
Remember, please give us your honest opinions and there are no right or wrong answers	
**ITALIAN TRANSLATION 1**	
Ricordi di dirci la sua sincera opinione, e che non ci sono risposte giuste o sbagliate.	
**ITALIAN TRANSLATION 2**	
Si ricordi, per favore, di darci la sua opinione sincera e che non ci sono risposte giuste o sbagliate	
**RECONCILED ITALIAN TRANSLATION**	

Esprima con sincerità le sue opinioni e ricordi che non ci sono risposte giuste o sbagliate.	
**BACKWARD TRANSLATION**	
Please answer as honestly as you can, and remember there are no right or wrong answers.	
**FINAL ITALIAN VERSION**	
Esprima con sincerità le sue opinioni e ricordi che non ci sono risposte giuste o sbagliate.	

Instruction 1, topic 1

ORIGINAL ENGLISH WORDING	COMMENTS/QUESTIONS
The following question asks about medical decision makers. A medical decision maker is a family member or friend who can make decisions for you if you were to become too sick to make your own decisions	
**ITALIAN TRANSLATION 1**	
Questa domanda riguarda il fiduciario per le decisioni mediche. Il fiduciario per le decisioni mediche è una persona, ad esempio un parente o un amico, autorizzata a prendere decisioni a suo nome nel momento in cui lei fosse troppo malato per decidere autonomamente.	
**ITALIAN TRANSLATION 2**	
La domanda seguente riguarda i decisori medici. Un decisore medico è un membro della famiglia o un amico che può prendere decisioni al suo posto nel caso lei diventasse troppo malato per prendere una decisione da solo.	
**RECONCILED ITALIAN TRANSLATION**	-
Questa domanda riguarda il fiduciario. Il fiduciario è una persona, ad esempio un membro della famiglia o un amico, che possa prendere decisioni a suo nome nel caso lei non fosse in grado di farlo	
**BACKWARD TRANSLATION**	

This question is about the surrogate decision-maker, who may, for example, be a family member or friend who can make decisions on your behalf if you are too sick.	
**FINAL ITALIAN VERSION**	
Questa domanda riguarda il fiduciario. Il fiduciario è una persona, ad esempio un membro della famiglia o un amico, che possa prendere decisioni a suo nome nel caso lei non fosse in grado di farlo.	

Instruction 2, topic 1

Chapter title

ORIGINAL ENGLISH WORDING	COMMENTS/QUESTIONS
READINESS	
**ITALIAN TRANSLATION 1**	
PREPARAZIONE	
**ITALIAN TRANSLATION 2**	
ESSERE PRONTO	
**RECONCILED ITALIAN TRANSLATION**	
GRADO DI PREPARAZIONE	
**BACKWARD TRANSLATION**	
DEGREE OF READINESS	
**FINAL ITALIAN VERSION**	
GRADO DI PREPARAZIONE	

TITLE topic 1

ORIGINAL ENGLISH WORDING	COMMENTS/QUESTIONS
Medical Decision Makers	

**ITALIAN TRANSLATION 1**	
Fiduciario per le decisioni mediche	
**ITALIAN TRANSLATION 2**	
Decisori medici	
**RECONCILED ITALIAN TRANSLATION**	
Il fiduciario	
**BACKWARD TRANSLATION**	
The surrogate decision-maker	
**FINAL ITALIAN VERSION**	
Il fiduciario	

Possible reply f)

ORIGINAL ENGLISH WORDING	COMMENTS/QUESTIONS
Not sure	
**ITALIAN TRANSLATION 1**	
Non è sicuro	
**ITALIAN TRANSLATION 2**	
Non (ne sono) sicuro	
**RECONCILED ITALIAN TRANSLATION**	
Non sa (cosa rispondere)	
**BACKWARD TRANSLATION**	
Not sure (what to answer)	
**FINAL ITALIAN VERSION**	
Non sa (cosa rispondere)	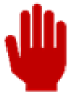 **QUERY # 3**: This response option could be presentalso in the self-assessed version ? (‘Non saprei’) + refuse (preferisco non rispondere)?

Possible reply g)

ORIGINAL ENGLISH WORDING	COMMENTS/QUESTIONS
Refused	
**ITALIAN TRANSLATION 1**	
Rifiuta di rispondere	
**ITALIAN TRANSLATION 2**	
(Mi sono) rifiutato	
**RECONCILED ITALIAN TRANSLATION**	
Rifiuta di rispondere	
**BACKWARD TRANSLATION**	
Refuses to answer	
**FINAL ITALIAN VERSION**	
Rifiuta di rispondere	**QUERY # 4**: This response option could be present also in the self-assessed version ? ('Preferisco non rispondere')

Additional question if reply e) was chosen

ORIGINAL ENGLISH WORDING	COMMENTS/QUESTIONS
OPTIONAL: If they answered, "I have already done it," then ask "When did you do this?"	
**ITALIAN TRANSLATION 1**	
OPZIONALE: Se la persona ha risposto "L'ho già fatto", chiedere "Quando l'ha fatto?"	
**ITALIAN TRANSLATION 2**	
ALTERNATIVA: se rispondono " L'ho già fatto" , allora domandare " Quando lo ha fatto?"	
**RECONCILED ITALIAN TRANSLATION**	
OPZIONALE: Se risponde "L'ho già fatto", chiedere "Quando l'ha fatto?"	

**BACKWARD TRANSLATION**	
OPTIONAL: If the reply is "I have already done it", ask "When did you do it?"	
**FINAL ITALIAN VERSION**	
OPZIONALE: Se risponde "L'ho già fatto", chiedere "Quando l'ha fatto?"	

Instruction 2, topic 2

ORIGINAL ENGLISH WORDING	COMMENTS/QUESTIONS
Please give us your honest opinions to the following questions about medical treatments. There are no right or wrong answers	
**ITALIAN TRANSLATION 1**	
Ci dica con sincerità la sua opinione sulle seguenti domande riguardo alle cure mediche. Non ci sono risposte giuste e risposte sbagliate.	
**ITALIAN TRANSLATION 2**	
Per favore ci dia la sua opinione sincera sulle seguenti domande in merito alle terapie mediche. Non ci sono risposte giuste o sbagliate.	
**RECONCILED ITALIAN TRANSLATION**	
Esprima la sua sincera opinione sulle seguenti domande riguardo ai trattamenti sanitari. Non ci sono risposte giuste o sbagliate.	
**BACKWARD TRANSLATION**	
Please reply as honestly as you can to the following questions about medical treatments. There are no right or wrong answers.	
**FINAL ITALIAN VERSION**	
Esprima la sua sincera opinione sulle prossime domande. Non ci sono risposte giuste o sbagliate.	

Chapter title, topic 2

ORIGINAL ENGLISH WORDING	COMMENTS/QUESTIONS
READINESS	
ITALIAN TRANSLATION 1	
PREPARAZIONE	
ITALIAN TRANSLATION 2	
ESSERE PRONTO	
RECONCILED ITALIAN TRANSLATION	
Grado di preparazione	
BACKWARD TRANSLATION	
DEGREE OF READINESS	
FINAL ITALIAN VERSION	
Grado di preparazione	

TITLE topic 2

ORIGINAL ENGLISH WORDING	COMMENTS/QUESTIONS
Care at EOL	
ITALIAN TRANSLATION 1	
ITALIAN TRANSLATION 2	
RECONCILED ITALIAN TRANSLATION	
BACKWARD TRANSLATION	
FINAL ITALIAN VERSION	When the grid was produced, this titled was skipped. For this reason, it was translated after the meetings
Cure nel fine vita	
