# Supplementary material for: Italian cross-cultural adaptation of the Quality of Communication questionnaire and the 4-item advance care planning engagement questionnaire
Source: PLoS One. 2023 Mar 23;18(3):e0282960. doi: 10.1371/journal.pone.0282960 (PMC10035811; doi:10.1371/journal.pone.0282960)
Supplement: S3 File — (PDF) [file pone.0282960.s003.pdf]

# S3 File – Cognitive interview guide
# QOC and 4- item ACP-Engagement

**Opening**

Thank you for your participation in the study. In a previous study phase, the questionnaire XX was translated into Italian from the original US English. In the present phase the translated questionnaire is debriefed with Italian adult patients to check whether it is well understood and accepted by the target audience. As a result, any contents (e.g., words, phrases, or concepts) that are unclear or inappropriate can be revised before large scale deployment of the questionnaire. In this interview we will assess your appraisal of the questionnaire as a whole, and considering each part. We will start from the introduction.
I apologize if the interview appears repetitive or becomes boring. We are interested in what you think about the questionnaire's contents and how you chose your answers to any given items. You can tell me any thoughts or opinion, whatever comes to your mind.
I'll share my screen / we'll look at the questionnaire together. I'll read each statement aloud – if you prefer, you can read it. I'll ask you whether each statement was clear, and, if not, your suggestions to make it clearer.

- **Looking at the introduction - What parts did you find unclear?**

**Comprehension**
- What does the statement mean to you, in your own words? (Probing questions: what does the word__mean to you? What do you understand by this sentence?Can you explain this sentence?)
- How easy or difficult was it to understand this item?
- (If there is an issue) what and how would you change?

**Retrieval**
- What did you think about answering this item? (Probing questions: was it easy or difficult? When you read this sentence, what are you thinking about?)

**Judgment**
- How confident were you in answering this item? (Probing question: How confident are you that you understood the purpose of this sentence?)
- Did all the response options make sense for this item?
- Is there anything else you would like to say about this item?

**Conclusion**
- What are your general opinions about the questionnaire?
- How could it be improved?
- Did you find any items/contents somehow inappropriate?
