# Supplementary material for: Italian cross-cultural adaptation of the Quality of Communication questionnaire and the 4-item advance care planning engagement questionnaire
Source: PLoS One. 2023 Mar 23;18(3):e0282960. doi: 10.1371/journal.pone.0282960 (PMC10035811; doi:10.1371/journal.pone.0282960)
Supplement: S4 File — (PDF) [file pone.0282960.s004.pdf]

File S4

Consolidated criteria for reporting qualitative studies (COREQ): 32-item checklist

No. Item	Guide questions/description	Reported on Page #
**Domain 1: Research team and reflexivity**		
Personal Characteristics		
1. Interviewer/facilitator	Which author/s conducted the interview or focus group?	LDP, MP, and MC. Pag. 8
2. Credentials	What were the researcher's credentials? E.g. PhD, MD	LDP: BSci, PhD<br>MP: BSci, PhD student<br>MC; BSci
3. Occupation	What was their occupation at the time of the study?	LDP: researcher<br>MP: PhD student,<br>MC: nurse.
4. Gender	Was the researcher male or female?	LDP: woman<br>MP: woman<br>MC: woman
5. Experience and training	What experience or training did the researcher have?	All the researchers had expertise in planning, conducting, and analyzing qualitative studies
Relationship with participants		
6. Relationship established	Was a relationship established prior to study commencement?	No relationship established prior to study commencement, pag. 9
7. Participant knowledge of the interviewer	What did the participants know about the researcher? e.g. personal goals, reasons for doing the research	No knowledge.
8. Interviewer characteristics	What characteristics were reported about the inter viewer/facilitator? e.g. Bias, assumptions, reasons and interests in the research topic	LDP is co-PI of the ConCure-SM study. MC is a member of the study's Steering Committee.
**Domain 2: study design**		
Theoretical framework		
9. Methodological orientation and Theory	What methodological orientation was stated to underpin the study? e.g. grounded theory, discourse analysis, ethnography, phenomenology, content analysis	Content analysis.
Participant selection		
10. Sampling	How were participants selected? e.g. purposive, convenience, consecutive, snowball	Purposive sampling, pag. 8
11. Method of approach	How were participants	By phone and email.

	approached? e.g. face-to-face, telephone, mail, email	
12. Sample size	How many participants were in the study?	9 patients, 3 significant others. Pag. 12, and Table 1.
13. Non-participation	How many people refused to participate or dropped out? Reasons?	Four patients and one significant other did not participate due to personal or organizational issues. One patient left the study before receiving the interview.
Setting		
14. Setting of data collection	Where was the data collected? e.g. home, clinic, workplace	Face-to-face, online, on the phone; Table 1
15. Presence of non-participants	Was anyone else present besides the participants and researchers?	No
16. Description of sample	What are the important characteristics of the sample? e.g. demographic data, date	Table 1
Data collection		
17. Interview guide	Were questions, prompts, guides provided by the authors? Was it pilot tested?	S3 File
18. Repeat interviews	Were repeat interviews carried out? If yes, how many?	No, they weren't.
19. Audio/visual recording	Did the research use audio or visual recording to collect the data?	The interviews were not audio-recorded, pag. 8.
20. Field notes	Were field notes made during and/or after the interview or focus group?	No, they weren't.
21. Duration	What was the duration of the inter views or focus group?	The interviews lasted between 26 and 60 minutes.
22. Data saturation	Was data saturation discussed?	No
23. Transcripts returned	Were transcripts returned to participants for comment and/or correction?	No
**Domain 3: analysis and findings**		
Data analysis		
24. Number of data coders	How many data coders coded the data?	Three data coders were involved, pag. 8
25. Description of the coding tree	Did authors provide a description of the coding tree?	No
26. Derivation of themes	Were themes identified in advance or derived from the data?	They have been derived from the data.
27. Software	What software, if applicable, was used to manage the data?	Not applicable
28. Participant checking	Did participants provide feedback	No.

	on the findings?	
Reporting		
29. Quotations presented	Were participant quotations presented to illustrate the themes/findings? Was each quotation identified? e.g. participant number	Table 2.
30. Data and findings consistent	Was there consistency between the data presented and the findings?	Yes (as study authors, we can be biased in the evaluation of this domain)
31. Clarity of major themes	Were major themes clearly presented in the findings?	Yes (as study authors, we can be biased in the evaluation of this domain)
32. Clarity of minor themes	Is there a description of diverse cases or discussion of minor themes?	No
