# Supplementary material for: Italian cross-cultural adaptation of the Quality of Communication questionnaire and the 4-item advance care planning engagement questionnaire
Source: PLoS One. 2023 Mar 23;18(3):e0282960. doi: 10.1371/journal.pone.0282960 (PMC10035811; doi:10.1371/journal.pone.0282960)
Supplement: Attachment — Submitted filename: Response to Reviewers.docx [file pone.0282960.s005.docx]

***Reviewer #1:***

*At the end of your introduction, state the objective of your research instead of what is written in the last paragraph “For each questionnaire, the linguistic validation encompasses two main actions: translation adaptation of the QOC and ACP-E inventories into Italian; psychometric validation. Here we report the results of the first action”.*

R: We have revised the last sentence of the introduction as suggested.

*The main aspect of the paper that can improve is the presentation of the cross-cultural adaptation (Results from page 9-12). Is it possible to create Tables to explain: what items were modified, why and how?*

R: We have added two tables to summarize the changes made in the QOC (Table 1) and 4-item ACP-E (Table 2).

*The conclusion should summarise your main results. Avoid citing references in your conclusion.*

R: We have revised the conclusion as suggested

***Reviewer #2:***

*The authors report that “For each questionnaire, the linguistic validation encompasses two main actions: translation adaptation of the QOC and ACP-E inventories into Italian; psychometric validation”. But they claim to report only the first part (i.e. Translation and adaptation). Why did the authors decide not to report the results of the psychometric properties?*

R: We purposely focused on a research phase that is usually poorly documented: translation and cultural adaptation (and cognitive debriefing). We have aired this in the discussion section.

*Even if the questionnaire has been transculturally translated, if we do not know the psychometric properties (such as reliability and validity) we cannot use it with our patients and therefore the repercussions in clinical practice are very low. I highly recommend reporting the psychometric results as well. Furthermore, I have hardly read of papers reporting only the assessment of cross-cultural validity; these results are usually reported together with the validity and reliability results, to improve the application of the results in clinical practice.*

R: The psychometric testing phase is currently ongoing and will be reported in a separate manuscript.

*The authors report that “the ACP-E is available in shorter versions (55-item, 34-item, 9-item, 4-item) … Specifically, we were interested in the 4-item version”. What are the reasons that led the authors to prefer the 4-item version? Why didn't they consider translating the entire completed questionnaire*?

R: We were interested in the 4-item version of the 4-item ACP-E as: (a) it had good sensitivity to change compared to longer versions in a large trial involving US English- and Spanish-speaking old adults (PREPARE study, ref. 12); (b) it assesses the readiness behavior change construct within the ACP ‘process’ domain (ref. 11); (c) administration burden is limited. We have described this in the materials and methods section.

*Information on the structure of the questionnaire is missing. For example, there is no information about the scores for each item and the calculation of the total score*

R: Following reviewer’s advice, we have added (methods section) the scoring rules for the two scales.

*The authors included only 14 patients in the cognitive de briefing. Typically, guidelines for cross-cultural validation suggest including 30 patients. Why did the authors only include 14 of them?*

R: As from the ISPOR Task Force, we applied cognitive debriefing, which involves a small group of 5-6 subjects. We guess that the reviewer refers to pilot testing instead, which requires 30–40 subjects.

***Reviewer #3: Peer-Review PONE-D-22-30497***

*Clinical relevance of the questionnaires*

*In the introduction, you explain about ACP - what it is, its elements etc. You also specify that the two questionnaires be used for clinical and research purposes (page 4, line 82/83). I would recommend being a bit more specific regarding the use cases you envisage. Are you translating the questionnaires for evaluation purposes of ACP interventions? Or are you planning to bring them into clinical practice? If yes, in which setting? How is shared decision making going to happen with the aid of these questionnaires?*

*You can also specify this in the discussion section, not in the background section if you felt it would sit better there.*

R: We have described (introduction section) that we are using the QOC and the 4-item ACP-E for evaluation purposes of an ACP intervention (ConCure-SM). Following reviewer’s advice, we have expanded the description of the ConCure-SM intervention and feasibility trial to clarify the context in which the two instruments are being used.

*Methods*

*You are very comprehensive when it comes to explaining your methods. Although you specify the methods for cognitive interviewing and you also specify the analysis methods, I was missing the framework of Tourangeau being applied to the data. From the results and how they are reported, I would expect you probably used Tourangeau. It might be worth mentioning him and his framework.*

R: We thank the reviewer for her comment. We do confirm that we pre-planned the interviews according to Willis' indications [14], and the modified Tourangeau model of cognitive aspects [15]. We have now slightly changed the text, accordingly.

*Discussion/article*

*Your aim is the cross-cultural adaptation of the two ACP questionnaires. You do a very thorough translation process. However, although cultural issues are implicitly mentioned in the results, the discussion section then does not make them explicit. You present a lot of evidence in the background on the Italian's readiness to endorse ACP. You also describe the health care system's lack in responding to this need. You describe the "emotionally taxing" nature of some of the items. I was wondering whether it would be possible to explicitly discuss cultural issues and how they reflect on the construct ACP the questionnaire want to measure. After all, achieving translations that are faithful to the original definition and measurement of the construct is one of the aims of a cross-cultural adaptation. The other might be to learn more about once culture and, in reflection, about the construct under investigation. Results from cross-cultural adaptations of questionnaires can feed back into further develop the content validity of the questionnaires. I was missing a section in the discussion to do just that - a bit of reflection on the general content validity of the two questionnaires.*

R: In the discussion section, we have now made explicit the main cultural issues that emerged during the cross-cultural adaptation process: “We also acknowledge that cultural issues arose during the study. These include a lack of knowledge of the Italian ACP legislation, revealed by the fact that some respondents were unaware of the term '*fiduciario*'. Another problem is the difficulty of talking about EOL in the Italian clinical context: although all enrolled patients suffered from progressive conditions, they expected their doctors to talk about possible treatments and care pathways, not about EOL. Death being a taboo topic, along with the superstitious belief that talking about something can evoke it, may contribute to this difficulty.”

*Minor comment*

*In the introduction section, you often speak of "his/her own personal values" or "his/her health". It is common to do a gender-neutral pronoun in plural in English in these situations (at least in publications, not in the actual questionnaires). Page 4, line 66 could better read as "ACP helps the patient to identify their own personal values and goals, understand their health..."*

R: We have changed the sentence as suggested.

*Otherwise, this was an engaging, clearly structured and methodologically thoroughly prepared study. What a pleasure to read!*

R: We thank the reviewer for her appreciation of the study.
